# Outcomes of early fiberoptic bronchoscopic sputum aspiration and lavage after thoracoscopic and laparoscopic esophageal cancer surgery: a randomized clinical trial

**DOI:** 10.1186/s13019-023-02370-7

**Published:** 2023-10-04

**Authors:** Wu Wang, Jin-biao Xie, Tian-bao Yang, Shi-jie Huang, Bo-yang Chen

**Affiliations:** Department of Cardiothoracic Surgery, Affiliated Hospital of Putian University, Putian, 351100 Fujian China

**Keywords:** Esophageal cancer, Fiberoptic bronchoscope, Lavage, Sputum aspiration, Thoracoscopic and laparoscopic esophagectomy, Complications

## Abstract

**Background:**

This study aims to investigate the outcomes of patients who received early fiberoptic bronchoscopic sputum aspiration and lavage after thoracoscopic and laparoscopic esophagectomy due to esophageal cancer.

**Methods:**

A prospective randomized clinical trial was performed between March 2020 and June 2022. Patients who were scheduled for thoracoscopic and laparoscopic esophagectomy due to esophageal cancer were enrolled. Then, these patients were assigned to the control group (traditional postoperative care) and study group (traditional postoperative care with early bronchoscopic sputum aspiration and lavage). The outcomes, which included the length of hospital stay and medical expenses, and postoperative complications, which included pulmonary infection, atelectasis, respiratory dysfunction and anastomotic leakage, were compared between these two groups.

**Results:**

A total of 106 patients were enrolled for the present study, and 53 patients were assigned for the control and study groups. There were no statistically significant differences in gender, age, and location of the esophageal cancer between the two groups. Furthermore, the length of hospital stay was statistically significantly shorter and the medical expenses were lower during hospitalization in the study group, when compared to the control group (12.3 ± 1.2 vs. 18.8 ± 1.3 days, 5.5 ± 0.9 vs. 7.2 ± 1.2 Chinese Yuan, respectively; all, *P* < 0.05). Moreover, there were statistically significantly fewer incidences of overall complications in study group, when compared to the control group (20.7% vs.45.2%, *P* < 0.05).

**Conclusions:**

For patients with esophageal cancer, early fiberoptic bronchoscopic sputum aspiration and lavage after thoracoscopic and laparoscopic esophagectomy can shorten the length of hospital stay, and lower the medical expense and incidence of postoperative complications.

## Background

Esophageal cancer has been reported as the 7th most common malignancy in the world [1]. The highest mortality rate of esophageal cancer was reported in patients residing in East Asia [1]. For patients with early localized esophageal cancer, the minimally invasive approach to thoracoscopic and laparoscopic esophagectomy has been shown to have comparable or better efficacy, when compared to traditional open surgery, with smaller surgical trauma, less blood loss, and faster postoperative recovery [2–4]. However, thoracoscopic and laparoscopic esophagectomy is commonly performed for patients in the prone or lateral decubitus position [5], which can result in increased postoperative pulmonary secretions. Furthermore, the pain and limited mobility of patients after surgery can restrict respiratory muscle movements and affect pulmonary functions, making it difficult to expectorate the secretions, and increasing the risk of postoperative complications, such as infection [6, 7]. Therefore, appropriate postoperative care is an indispensable part of comprehensive management, in order to achieve satisfactory recovery.

It has been reported that different strategies, including adequate pain control, respiratory muscle training, nebulizer treatment, fluid balance control, and corticosteroid administration, can be employed to decrease pulmonary secretions after esophagectomy, with variable efficacies [8–10]. Furthermore, oral or nasal suctioning can be performed to remove abundant secretions, but its blind use might cause mucosal damage and infection [11]. Moreover, bronchoscopy is a commonly used diagnostic procedure [12]. Clinical trials have revealed that bronchoscopic sputum aspiration can improve the outcomes of patients with acute exacerbation of chronic obstructive pulmonary disease [13, 14]. Another randomized clinical trial also reported that the severe lung infection improved after bronchoscopic lavage [15]. Based on these evidences, the investigators considered whether early fiberoptic bronchoscopic sputum aspiration and lavage after thoracoscopic and laparoscopic esophagectomy can benefit the surgical recovery of patients with esophageal cancer.

The present study conducted a randomized clinical trial, and compared the postoperative outcomes of patients with or without fiberoptic bronchoscopic sputum aspiration and lavage after minimally invasive thoracoscopic and laparoscopic esophagectomy, with the purpose of improving the recovery and prognosis of these patients.

## Methods

### Study design and participants

A prospective randomized clinical trial was performed at the Affiliated Hospital of Putian University, Putian, Fujian, China, between March 2020 and June 2022. The study protocol was approved by the hospital ethics committee. All study participants provided a signed informed consent.

The participant inclusion criteria were, as follows: (1) patients between 44 and 76 years old; (2) esophageal cancer patients who were scheduled for thoracoscopic and laparoscopic esophagectomy; (3) patients with normal preoperative cardiopulmonary, hepatic and renal function evaluations. The exclusion criteria were, as follows: (1) patients with a previous history of psychiatric disorder, (2) patients with coagulation dysfunction, and (3) patients with previous thoracic surgical operations.

### Study protocol

The baseline characteristics, including the demographics, history of smoking, alcohol abuse, diabetes, chronic obstructive pulmonary disease (COPD), neoadjuvant therapy, and location and stage of the esophageal cancer, were recorded. The patients were randomized to the control and study groups using the random ball method. After the thoracoscopic and laparoscopic esophagectomy, patients in the control group received traditional treatment, while patients in the study group received fiberoptic bronchoscopic lavage and sputum aspiration, in addition to traditional treatment.

The thoracoscopic and laparoscopic esophagectomy was performed according to the standard method [16]. At post-operation, the patients were transferred to the post-anesthesia care unit. Then, when the patients were fully awake and stabilized, they were transferred back to the medical ward. Patients in the control group received traditional postoperative respiratory care, which included deep breathing exercise training, albuterol nebulization, nasal aspiration, and back clapping, in order to facilitate sputum expectoration. In the study group, in addition to the traditional postoperative respiratory care, the patients also received early fiberoptic bronchoscopic sputum aspiration and lavage once a day on postoperative day 1, 3 and 5.

The fiberoptic bronchoscopic sputum aspiration and lavage was performed according to the following steps: (1) the fiberoptic bronchoscope, adequate light source system, negative pressure suction, oxygen saturation monitor, oxygen supply, and cardiac monitor were prepared in advance; (2) the patient was laid flat, and high-flow oxygen (6–8 L/min) was provided for 20 min before the procedure; (3) the nasal cavity was cleaned, and topical 2% lidocaine was applied for local anesthesia; (4) a fiberoptic bronchoscope was inserted through one nostril, and high-flow oxygen was provided through the other nostril to maintain an oxygen saturation of > 90% throughout the procedure; (5) a bronchoscope was entered into the left and right bronchus, respectively, and the sputum was aspirated with a constant negative pressure of 150 mmHg (if the patient had thick sputum, 10–20 ml of saline was used for repeated flushing and aspiration, and the lavage fluid was sent for culture and sensitivity analysis; if there was a laboratory positive result for confirmed bacterial infection and pulmonary infection, alveolar lavage was performed with the relevant sensitive antibiotics); (6) the fiberoptic bronchoscope was withdrawn. The whole procedure was performed for approximately 15 min. The procedure was terminated when the patient had bradycardia, hypotension, or an oxygen saturation of < 85%.

### Outcome and postoperative complication measurements

The outcomes were the entire length of hospital stay and the medical expenses during hospitalization. Postoperative complications, including pneumonia, atelectasis, pulmonary dysfunction, vocal cord paralysis, and anastomotic leakage, were documented. Atelectasis was defined as the partial or complete collapse of one side, lobe, or segment of the lungs. Pulmonary dysfunction was defined as an arterial blood oxygen partial pressure of < 60 mmHg in the resting position in room air, with or without a carbon dioxide partial pressure of > 50 mmHg, after excluding interventricular shunt and reduced cardiac output. Vocal cord paralysis refers to the loss of adduction, abduction, and normal tone of the vocal cords, resulting in voice change, protective laryngeal sphincter adduction, or difficult breathing. It was diagnosed by the fiberoptic bronchoscopic examination in patients with clinical symptoms suspected to have vocal cord injury. Anastomotic leakage is defined as the formation of a fistula due to an unsealed or poorly healed anastomosis between the esophagus and stomach.

### Statistical analysis

Continuous data were presented as mean ± standard deviation, and compared using Student *t*-test. Categorical data were presented in number with percentage, and compared by performing Chi-square analysis. All statistical analyses were performed using SPSS (version 25.0; IBM, NY, USA). A *P*-value of < 0.05 was considered statistically significant.

## Results

### Patient enrollment and comparison of baseline characteristics

**Fig. 1:**
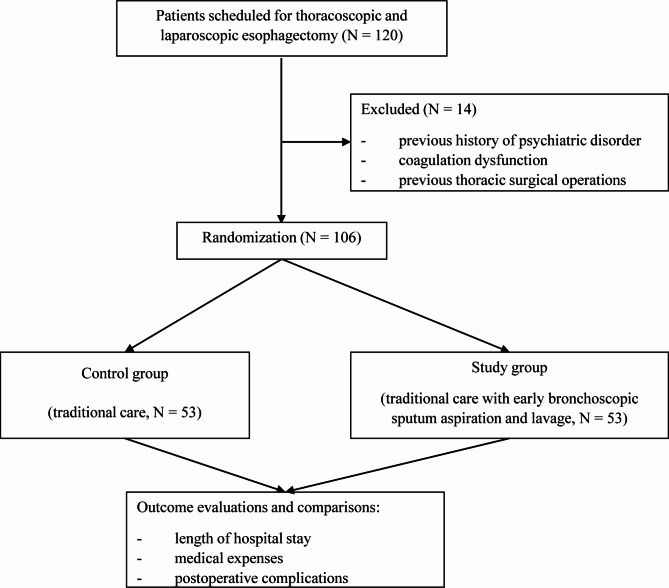
CONSORT flow diagram

A total of 106 patients were enrolled and completed the study. Then, 53 patients were assigned to the control and study groups (Fig. 1). All patients successfully completed the thoracoscopic and laparoscopic esophagectomy. None of the bronchoscopic procedures was terminated. There were no statistically significant differences in gender, age, history of smoking, alcohol abuse, neoadjuvant therapy, COPD, diabetes, and location and stage of the esophageal cancer between the two groups (Table 1).

**Table 1:** Comparison of baseline characteristics between the two groups

Characteristics	Study group (*n* = 53)	Control group (*n* = 53)	*P*
Gender, *n* (%)			> 0.05
Male	41 (77.4%)	40 (75.5%)	
Age, year, mean ± standard deviation	59.2 ± 3.9	59.2 ± 3.9	> 0.05
Smoking, *n*	17	12	> 0.05
Alcohol abuse, *n*	5	6	> 0.05
COPD, *n*	14	11	> 0.05
Diabetes, *n*	10	12	> 0.05
Neoadjuvant therapy, *n*	4	6	> 0.05
Location of cancer, *n* (%)			
Upper	3 (5.7%)	4 (7.5%)	> 0.05
Middle	39 (73.6%)	36 (67.9%)	> 0.05
Lower	11 (20.8%)	13 (24.5%)	> 0.05
Cancer stage, I/II/III/IV, *n*	17/9/24/3	19/13/19/2	> 0.05

COPD, chronic obstructive pulmonary disease

### Comparison of outcomes between the two groups

Compared to patients in the control group, patients in the study group had a shorter length of hospital stay, and lower medical expenses during hospitalization (Table 2).

**Table 2:** Comparison of outcomes between the two groups

Outcomes	Study group (*n* = 53)	Control group (*n* = 53)	*P*
Length of hospital stay, days	12.3 ± 1.2	18.8 ± 1.3	< 0.05
Medical expenses, 10,000 RMB	5.5 ± 0.9	7.2 ± 1.2	< 0.05

RMB, Renminbi (Chinese Yuan, CNY). All data were presented in mean ± standard deviation

### Comparison of postoperative complications between the two groups

Compared to patients in the control group, patients in the study group had lower overall incidences of complications (45.2% vs. 20.7% for the control and study groups, respectively; Table 3). Furthermore, the respiratory complications (pneumonia, atelectasis, and pulmonary dysfunction) were three (5.7%) and 15 (28.3%), respectively (*P* < 0.05). Moreover, patients with vocal cord paralysis or COPD were further analyzed. It was found that the incidence of pneumonia was statistically and significantly lower in the study group, when compared to the control group (Table 4).

**Table 3:** Comparison of postoperative complications between the two groups

Postoperative complications, *n* (%)	Study group (*n* = 53)	Control group (*n* = 53)	*P*
Overall	11 (20.7%)	24 (45.2%)	< 0.05
Pneumonia	2 (3.8%)	10 (10.9%)	< 0.05
Atelectasis	1 (1.9%)	3 (5.7%)	> 0.05
Pulmonary dysfunction	0 (0.0%)	2 (3.8%)	> 0.05
Vocal cord paralysis	7 (13.2%)	6 (11.3%)	> 0.05
Anastomotic leakage	1 (1.9%)	3 (5.7%)	> 0.05

**Table 4:** Comparison of pneumonia incidences in patients with vocal cord paralysis or chronic obstructive pulmonary disease between the two groups

	Study group (*n* = 53)	Control group (*n* = 53)	*P*
Patients with pneumonia/Patients with vocal cord paralysis, *n*/*n* (%)	1/7 (14.3%)	4/6 (66.7%)	< 0.05
Patients with pneumonia/Patients with COPD, *n*/*n* (%)	2/14 (14.3%)	6/11 (54.5%)	< 0.05

COPD, chronic obstructive pulmonary disease

## Discussion

The minimally invasive approach to thoracoscopic and laparoscopic esophagectomy has been applied for patients with esophageal cancer. The successful management of respiratory secretion can decrease postoperative complications and improve patient outcomes. The present study performed early fiberoptic bronchoscopic sputum aspiration and lavage after thoracoscopic and laparoscopic esophagectomy. The present study results revealed that patients had a shorter length of hospital stay, and lower medical expenses and incidences of postoperative complications. Early fiberoptic bronchoscopic sputum aspiration and lavage can be used after thoracoscopic and laparoscopic esophagectomy to improve patient outcomes.

For patients with esophageal cancer, thoracoscopic and laparoscopic radical esophagectomy can preserve the latissimus dorsi and intercostal muscles, in order to maintain respiratory function and facilitate postoperative recovery [17]. However, the duration of the surgical operation is usually long, and patients are placed in the prone or lateral decubitus position, causing the accumulation of airway secretions and fluid [18]. Furthermore, the surgical procedure in the esophagus might injure the surrounding structures, such as the diaphragm muscle, phrenic nerve and recurrent laryngeal nerve, reducing the cough reflex, and affecting respiratory function [19]. Moreover, during the surgery, the stomach is lifted from the abdominal cavity into the thoracic cavity, which could compress the lungs and limit the respiration. In addition, most patients with esophageal cancer are middle-aged or elderly people with multiple underlying medical conditions. Thus, the sedation caused by the intraoperative anesthetic medications, the tracheal mucosal injury caused by the intubation, and the postoperative pain can affect the patient’s respiratory function, reduce the patient’s ability to expectorate sputum, and cause pulmonary complications, which can lead to serious outcomes for affected patients. Pulmonary infection is a serious postoperative complication in patients with esophageal cancer [20]. Serious pulmonary infections can cause death. Prompt postoperative management to remove excessive airway secretions and fluid can prevent pulmonary infection, and improve the successful outcome of the surgery.

The traditional postoperative care to facilitate sputum expectoration include oral or nasal suction, nebulizer treatment, breathing exercise, back clapping, and corticosteroid administration [8–10]. These methods might help to decrease the secretion and spit the sputum, but can lead to variable results in different patient populations. For example, elderly patients might not be able to participate in breathing exercises or well-tolerate back clapping after a major surgery. Early fiberoptic bronchoscopic sputum aspiration is performed using a fiberoptic bronchoscopic catheter to remove airway secretions at the early postoperative stage. During the bronchoscopy, the physician can directly inspect the bronchi. If thick sputum and secretion are found, lavage can be used to remove these. Then, the aspirated secretion or lavage fluid can be sent for bacterial culture and sensitivity analysis. When a suspicious or confirmed infection is found, sensitive antibiotics can be locally applied to have adequate coverage. Pulmonary lavage can stimulate the local airway to directly induce cough reflex, leading to improved therapeutic effects.

In the present study, patients in the study group had a shorter length of hospital stay, when compared to patients in the control group. Furthermore, patients in the study group incurred lower medical expenses. All these were attributed to the successful sputum removal and pulmonary lavage through the early fiberoptic bronchoscopic procedure. In addition, antibiotics were locally injected for these patients to reach a high topic concentration of antibiotics in the pulmonary tissues. All these can shorten the length of hospital stay and reduce medical expenses, ultimately improving patient outcomes.

The incidence of postoperative complications was also investigated in the present study. The results revealed that the incidence of overall complications was 45.2% and the incidence of respiratory complications was 28.3% in the control group. The respiratory complication was lower than that reported by a previous study (40% respiratory complication) [21]. Furthermore, patients in the control group had a lower incidence of postoperative anastomotic leakage, when compared to the incidence reported by a previous study [22]. These might be due to the difference in patient population, cancer stage, and postoperative care. The present study revealed that early fiberoptic bronchoscopic treatment can further decrease the incidence of overall complications and anastomotic leakage. In addition, the further analysis of patients with vocal cord paralysis or COPD revealed a lower incidence of pneumonia in patients in the study group, when compared to patients in the control group. All these evidence suggested that early fiberoptic bronchoscopic aspiration lavage can directly reach the lung segment and its bronchi at all levels, and remove mucous secretions from the airway under direct vision. However, the lavage can dilute the inflammatory exudates, which can cause damage to the airway mucosa, and stimulate the local airway mucosa. The bronchoscopic treatment might improve the physiological response of the body to cough, and allow for the easy expectoration of secretions, decreasing the incidence of complications.

The limitations of the present study include the single-center nature of the study, and the small sample size. Furthermore, the performance of the fiberoptic bronchoscopy and sputum removal was influenced by the skill of the operator. The present study results need to be confirmed through future large-scale studies.

## Conclusions

In conclusion, early fiberoptic bronchoscopic sputum aspiration and lavage after thoraco-laparoscopic esophagectomy can shorten the length of hospital stay, and lower the medical expenses and incidence of postoperative complications of patients with esophageal cancer. However, further studies are warranted.

## Data Availability

The datasets used and/or analyzed during the study are available from the corresponding author on reasonable request.
